# A Paper-Based Photoelectrochemical Sensing Platform Based on In Situ Grown ZnO/ZnIn_2_S_4_ Heterojunctions onto Paper Fibers for Sensitively Detecting AFP

**DOI:** 10.3390/bios12100818

**Published:** 2022-10-02

**Authors:** Jiali Huang, Xu Li, Mingzhen Xiu, Kang Huang, Kang Cui, Jing Zhang, Shenguang Ge, Shiji Hao, Jinghua Yu, Yizhong Huang

**Affiliations:** 1 School of Chemistry and Chemical Engineering, University of Jinan, Jinan 250022, China; 2 School of Materials Science and Engineering, Nanyang Technological University, Singapore 639798, Singapore; 3 Institute for Advanced Interdisciplinary Research, University of Jinan, Jinan 250022, China; 4 School of Materials Science & Engineering, Dongguan University of Technology, Dongguan 523808, China

**Keywords:** *μ*PAD, photoelectrochemical, alpha-fetoprotein, point-of-care assay, immunosensing

## Abstract

Nowadays, developing a cost-effective, easy-to-operate, and efficient signal amplification platform is of important to microfluidic paper-based analytical devices (*μ*PAD) for end-use markets of point-of-care (POC) assay applications. Herein, an ultrasensitive, paper-based photoelectrochemical (PEC) bioassay platform is constructed by in situ grown ZnO/ZnIn_2_S_4_ heterojunctions onto paper fibers, which acted as photoactive signal amplification probes for enhancing the sensitivity of antibodies-based diagnostic assays, for the sensitive detection of alpha-fetoprotein (AFP) targets. The crystalline flake-like ZnIn_2_S_4_ composited with hexagonal nanorods (NRs) morphology of ZnO is an in situ grown, at the first time, onto cellulose fibers surface supported with Au nanoparticle (Au NP) modification to improve conductivity of the device working zone. The obtained composites on paper fibers are implemented as a flexible paper-based photoelectrode to realize remarkable performance of the fabricated *μ*PAD, resulting from the enhanced PEC activity of heterojunctions with effective electron-hole pair separation for accelerating photoelectric conversion efficiency of the sensing process under light irradiation. Once the target AFP was introduced into the biosensing interface assistant, with a specific recognition interaction of AFP antibody, a drastically photocurrent response was generated, in view of the apparent steric effects. With the concentration increase of AFP targets, more immune conjugates could be confined onto the biosensing interface, eventually leading to the quantitative decrease of photocurrent intensity. Combined with an ingenious origami design and permitting the hydrophobic/hydrophilic conversion procedure in the bioassay process, the ultrasensitive PEC detection of AFP targets was realized. Under the optimized conditions, the level of AFP could be sensitively tracked by the prepared *μ*PAD with a liner range from 0.1 to 100 ng mL^−1^ and limit of detection of 0.03 ng mL^−1^. This work provides a great potential application for highly selective and sensitive POC testing of AFP, and finally, developments for clinical disease diagnosis.

## 1. Introduction

Recently, developing accurate, reliable, and user-friendly sensing devices for point-of-care (POC) assay applications is one of the most important objectives in clinical diagnosis [1,2,3]. This is based on the fact that, in any remote locations, an available POC diagnostic device is extremely vital to aid disease diagnosis and treatment selections. Normally, POC assay devices with obvious advantages, such as low-cost, ease of use, and real-time monitoring, are designed to be portable for fewer resources requirement than clinical diagnosis [4,5]. To meet the criterion in demanded for cost-effective POC diagnostic devices, various substrates with advantage of macro- and mesoporous structures, ease of folded manufacturing, and well-established surface modification, have been widely explored [6,7,8]. Among them, a microfluidic paper-based analytical device (*μ*PAD) has become one of the most generally employed POC assay devices applied in many areas, such as, pregnancy testing, drug abuse, and blood infection diagnoses, which is attribute to the intrinsic merits of cellulose paper, including easy functionalization, good biodegradability, and acceptable biocompatibility, in the construction of portable diagnostic devices [9,10,11,12,13]. So far, regarding the amount of appealing properties of paper substrate, the *μ*PAD integrated with different analytic technologies, such as colorimetric, electrochemiluminescence, and fluorescence, have been successfully exploited to produce paper-based colorimetric biosensing platform, enzyme-based paper biosensing platform, paper-based electrochemical biosensing platform, and even self-powered paper-based analytical device for revolutionizing clinic diagnostics [14,15,16,17,18,19,20]. To realize it, by taking advantage of unique properties of cellulose paper, various nanomaterials with certain functional properties can be subtly modified onto the paper fibers surface, which is regarded as one of the most key aspects in the development of highly effective signal approaches for fitting the bill as an out-bound substrate to fabricate this kind of portable biosensing devices [21,22,23,24,25].

Alpha-fetoprotein (AFP), as one of the most basilic tumor biomarkers, with a molecular weight about 70,000 Da, is normally used for routine cancer screening in clinical laboratories because it is universally associated with many cancer incidences, including gastric, breast, colorectal, and liver cancers [26,27,28,29]. Accordingly, it is valuable to explore sensitive, accurate, and effective bioassays strategies in biological samples combined with various techniques for AFP determination. Immunoassays, due to their highly biospecific interactions from antibodies/antigens recognition, play a key role in the development of analytical techniques for the quantitative determination of tumor biomarkers [30,31]. With these considerations, it is highly reasonable to develop a paper-based immunoassay strategy for the accurate quantification of AFP targets, in order to meet the pressing need of practical diseases diagnosis, especially in resource-limited regions, where the medical infrastructures are limited. To date, approaches such as electrochemiluminescence, electrochemical, and fluorescence have been proposed for the timely detection of AFP [32,33,34,35]. There is no doubt that those approaches have made significant progress in the fields of clinical applications. However, many such proposed detection methods, with related diagnostics techniques, still meet the challenge of the clinical applications, due to the high time consumption, low detection efficiency, and expensive diagnostic instruments. Accordingly, there is a great concern and pressing need to construct more sensitive, cost-effective, and efficient methods for practical AFP quantification of clinical laboratories.

To address the concerns, paper-based photoelectrochemical (PEC) analytical devices, which combined the advantage of PEC techniques and intrinsic merits of cellulose paper, have presented potential advantages in clinical applications, including device portability, facile operation, and high sensitivity, with a low signal background [36,37,38,39]. Compared with that of a traditional electrochemical detecting scheme, where a special potential needs to be applied to produce the readout signals, the sensing process of PEC is able to satisfactorily reduce the bias dependence on the signal generation own to the improved redox properties of photoactive nanomaterials, with their efficient carrier separation capability. Furthermore, two complete independence parts for excitation source (light) and sensing signal (electricity) are designed in sensing scheme of PEC, potentially exhibiting a higher sensing technique, due to its reduced signal background noise [40,41,42]. Regarding the requirement of efficiently enhancing PEC device performance, the exploration of photoactive materials is always actively pursued in the field of fabrication, as it plays a key role in the photoelectric signal conversion in the PEC sensing process, resulting from the efficient charge transfer and electron-hole pair separation of the prepared photoactive nanomaterials, under corresponding light irradiation. Besides, an elaborate structure design with intrinsic property improvement has also been pursued by scientists to achieve sensitive PEC bioassay via varied photoactive materials, such as carbon-nanomaterials, perovskite nanomaterials, and organic-inorganic hybrid nanomaterials [43]. Generally, a semiconductor photoanode with enhanced visible light-harvesting ability is able to suppress charge recombination in view of easily facilitating the separation of electron-hole pairs.

As a typical semiconductor, ZnO, with its admirable photoelectric properties and excellent chemical stabilities, has been widely utilized as an effective photoactive nanomaterial to construct a device for PEC bioassay applications [44,45,46,47,48]. On the other hand, the intrinsic broad band gap (3.37 eV) of electronic and undesired rapid recombination rate of photo-induced electron/hole pairs of ZnO have highly limited its photocurrent conversion efficiency, further inducing the reduced photo-current signal during PEC bioassay process. To solve this issue, one of efficient approaches is vacancy engineering, based on the fabrication of binary heterojunctions by hybridizing ZnO, with semiconductors possessing a narrow bandgap for enhancing the ZnO PEC performance [40,49,50,51]. ZnIn_2_S_4_, as a typical ternary semiconductor chalcogenide material, has drawn considerable attention, in the context of PEC bioanalysis and biosensors, on account of its proper band gap (2.34 eV–2.48 eV), non-toxic property, and lack of susceptibility to destruction from photocorrosion [52,53,54]. Consequently, it is decidedly desirable to construct a heterojunction between ZnO and ZnIn_2_S_4_ to expand the light absorption spectrum, improve the electron transfer efficiency, and accelerate photogenerated electron-hole separation and, thus, the enhancing energy conversion efficiency. Combined with the unique merits of cellulose paper, such as loose structure and muricate surface, it is highly desirable for the construction of ZnO/ZnIn_2_S_4_ heterojunction onto paper fibers for enhancing the performance of paper-based PEC analytical devices [55].

**Scheme 1:**
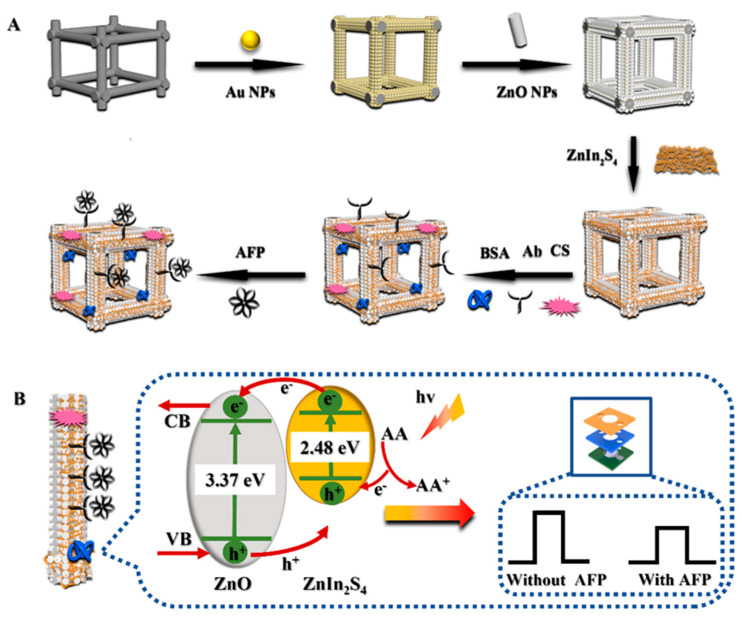
(**A**) Construction procedures of the proposed paper-based PEC bioassay platform. (**B**) Schematic diagram for the PEC biosensing illustration of *μ*PAD.

Herein, we present an ultrasensitive PEC *μ*PAD enhanced with the photoactive signal amplification probes of ZnO/ZnIn_2_S_4_ heterojunctions onto the modified paper fibers to successfully achieve the highly sensitive detection of AFP. The hexagonal nanorods (NRs) of ZnO, composited with crystalline flake-like ZnIn_2_S_4_, were grown in situ onto the modified paper fibers, of which, the Au nanoparticle (Au NP) layer was utilized to improve the conductivity, the first time, and significantly boost the paper electrode active area and facilitate the electron extraction from heterojunction interface. In this case, the built-in electric field of interface between ZnIn_2_S_4_ and ZnO could effectually strengthen the photoexcited electron/hole pairs conversion efficiency, thereby causing the descent of the photocurrent signal. As elucidated in Scheme 1A, to further realize the PEC biosensing, the AFP antibodies (Ab) were anchored onto the prepared ZnO/ZnIn_2_S_4_ heterojunctions at the Au NP-modified paper working electrode (Au-PWE), with the assistance of glutaraldehyde (GLD) and chitosan (CS) to bind the AFP. Then, the AFP can be specifically tied up onto the surface of flexible paper-based photoelectrode via the specific recognition of antigen-antibody, with the help of the introduction of bovine serum albumin (BSA) for staying away from any possible nonspecific interaction. In this situation, the increase of AFP concentration induces the quantifiable decrease of photo-current signal response from the constructed paper-based photoelectrode. It is caused by the reduced electron donor consumption of ascorbic acid (AA) and distinct steric hindrance during the PEC bioassay process. As a result, the AFP target-dependent immunoassays could be realized by the measurement of the photocurrent for the accurate quantification of AFP targets. In addition, on the basis of a subtle design of *μ*PAD, the flexible paper-based photoelectrode of the proposed *μ*PAD was developed to detect the human-blood serum sample, thus elucidating a tremendous potential application in the exploration of POC diagnostic devices for clinical disease diagnosis.

## 2. Materials and Methods

### 2.1. Chemicals and Reagents

Aqueous solutions of all experiments were prepared with ultrapure water. All reagents were received without being further purified. Thioacetamide (TAA), ZnCl_2_, InCl_3_·4H_2_O, ascorbic acid (AA), and K₃ [Fe (CN)₆] were supplied from Aladdin (Shanghai, China). Zn (NO_3_)_2_·6H_2_O were received from Damao Chemical Reagent Company (Tianjin, China). Hexamethylenetetramine was purchased from Sinopharm Chemical Reagent Co., Ltd. (Shanghai, China). Chitosan (CS) was purchased from Solarbio Co., Ltd. (Beijing, China). Glutaraldehyde (GLD; 50%) was ordered from Tianjin Chemical Plant Co., Ltd. (Tianjin, China). Na_2_HPO_4,_ KH_2_PO_4_, KCl, bovine serum albumin (BSA), alpha-fetoprotein (AFP) antigen, and antibody (Ab) were obtained from Sigma-Aldrich Chemical Co. Ltd. (Shanghai, China). Chloroauric acid (HAuCl_4_·4H_2_O), carcinoembryonic antigen (CEA), prostate-specific antigen (PSA), and squamous cell carcinoma antigen (SCC) were obtained from Shanghai Linc-Bio Science Co., Ltd. (Shanghai, China). Human immunoglobulin G (H-IgG) and human chorionic gonadotropin (HCG) were ordered from Shanghai Sangon Biotech Co., Ltd. (Shanghai, China).

### 2.2. Apparatus

The PEC signals and electrochemical impedance spectroscopy (EIS) were measured using a typical CHI 660C electrochemical working station via a conventional three-electrodes cell. ZnO/ZnIn_2_S_4_ photoelectrode was prepared as the working electrode. Ultroviolet-visible (UV-vis) optical absorption spectra was performed via a Schimidzu UV-1800. Scanning electron microscopic (SEM) and the energy-dispersive spectrum (EDS) elemental mapping measurements were characterized via a QUANTA FEG 250 SEM (Oxford Co., Oxford, UK). Infrared spectras were measured with a Fourier transformed infrared resonance (FT-IR) spectrum RX (PerkinElmer Spectoment). X-ray diffraction (XRD) patterns were recorded by a D8 advanced diffractometer system, equipped with Cu Kα radiation (Bruker Co., Bremen, Germ). X-ray photoelectron spectroscopy (XPS) spectra were acquired with an ESCALAB MK II X-ray photoelectron spectrometer.

### 2.3. Sensing Mechanism of μPAD

The mechanism of the proposed PEC *μ*PAD, based on in situ growth of ZnO/ZnIn_2_S_4_ heterojunctions on paper fibers, as a probe to amplify PEC signals, is shown in Scheme 1B. Firstly, with electrodeposition strategy, the vertical distribution of ZnO was modified onto the Au/paper substrate, which was for the preparation of paper-based ZnO. Then, the crystalline flake-like ZnIn_2_S_4_, with narrow bandgaps, were further grown on paper-based ZnO by a simple one-step hydrothermal method to form a paper-based ZnO/ZnIn_2_S_4_ heterojunction. Compared with ZnO alone, ZnO/ZnIn_2_S_4_ heterostructure enhances the absorption capacity of light source, so as to significantly improve the utilization rate of sunlight energy with the enlarged absorption range of light. Due to suitable band structure, the binary composites (ZnO/ZnIn_2_S_4_) could form type-Ⅱ heterojunction that can effectively enhance the efficiency of photogenerated carrier separation. The heterojunction could enhance photogenerated charge carries separation: the electronic (e^−^) on the valence band (VB) of ZnIn_2_S_4_ could jump to conduction band (CB) by light illumination and flow to CB of ZnO, due to the effect of electrostatic field. The excessive photogenerated hole (h^+^) could accumulate on the CB of ZnIn_2_S_4_ and be consumed by AA of solution. As for the whole mechanism of *μ*PAD detection of AFP: the AFP is introduced on the surface of photoactive material through specific reaction with Ab; after that, AFP not only impedes the transition of photogenerated carries, but also influences the consumption of h^+^, due to the inherent poor conductivity and space effect, thus realizing the PEC detection of AFP.

### 2.4. Preparation of the Proposed μPAD

#### 2.4.1. Electrodeposition of Paper-Based ZnO

Firstly, the Au NPs layer was grown onto the paper working zone of the *μ*PAD to improve the electrical conductivity of cellulose fibers (the details were introduced in Supporting Information). Then, the hexagonal NRs morphology of ZnO were grown in situ onto the Au NP-modified paper working electrode (Au-PWE) of the *μ*PAD by electrodepositing method at −0.8 V for 7200s, within solution dissolved 0.16 mM Zn (NO_3_)_2_·6H_2_O and 0.1 mM hexamethylenetetramine [56]. A frequently used, three-electrode system, containing working (a 0.95 cm^2^ circular area paper electrode modified with the Au NPs layer), counter (Pt wire), and reference (Ag/AgCl) electrodes, was adopted to realize electrodeposition of hexagonal NRs ZnO onto paper working zone. Finally, the obtained paper-based ZnO was rinsed thoroughly with distilled water and dried at 60 ℃ for further usage.

#### 2.4.2. Fabrication of Paper-Based ZnO/ZnIn_2_S_4_ Photoelectrode

The growth of the layer of crystalline flake-like ZnIn_2_S_4_ on the paper-based ZnO by a solvothermal method is based on a protocol with a little modification [57]. Briefly, ZnCl_2_ (0.17 mmol, 0.023 g) and InCl_3_·4H_2_O (0.33 mmol, 0.074 g) were dissolved in 30 mL ultrapure water, under even stirring for 0.5 h. Afterwards, thioacetamide (TAA, 0.667 mmol, 0.050 g) was added to the just-mentioned liquid, stirring again. The obtained sample was then shifted into two 50 mL Teflon-lined stainless-steel autoclaves, in which the prepared paper-based ZnO was immersed using the modificatory side face down. Subsequently, the autoclaves were hydrothermally treated at 150 °C in an electric oven for 1 h. Then, fully cooled, the paper-based ZnO/ZnIn_2_S_4_ photoelectrodes were taken out, washed via ethanol and ultrapure water, and fully dried at 60 °C.

#### 2.4.3. Practical Structural Design and Analytical Steps of the *μ*PAD

**Figure 1:**
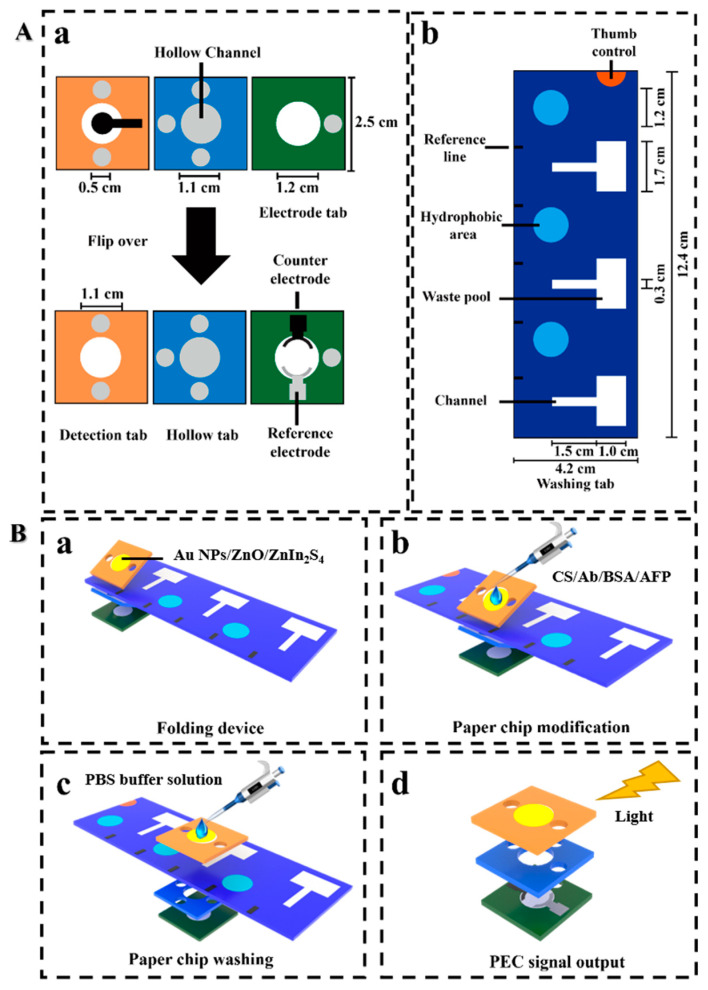
(**A**) The specific size and design of the *μ*PAD: (a) Detection tab, Hollow tab, and Electrode tab. (b) Washing tab. (**B**) Modification and PEC detection of the *μ*PAD: (a) Folding device. (b) Paper chip modification. (c) Paper chip washing. (d) PEC signal output.

The characteristic patterns of *μ*PAD were designed by Adobe Illustrator CS4 and printed on Whatman No. 1 chromatography paper using a wax printer (Figure S1, Supplementary Materials). The wax-printed paper devices need to bake at 200 °C for 30 s to melt the wax and form a 3D hydrophobic wall. Figure 1A shows the overall design and size of the proposed *μ*PAD, which consists of detection (the yellow tab, including a circle 1.1 cm in diameter and two holes 0.5 cm in diameter), hollow (the blue tab, including a hole 1.1 cm in diameter and three holes 0.5 cm in diameter), electrode (the green tab, including a circle 1.2 cm in diameter, which printed counter electrode and reference electrode, as well as a hole 0.5 cm in diameter), and washing (the dark blue tab consists of thumb control, reference line, hydrophobic area, and waste pool (including two rectangles)) tabs. The large and small grey circles in each tab are cut off by scissors to form holes, in order to realize the connection of three-electrode system, when the electrolyte solution is added, and further making it easier for the three electrodes to connect with electrochemical workstation. The design of washing tab is shown, in detail, in Figure 1A, where the clever alternating arrangement of hydrophobic tabs with waste pools can perfectly handle the excess waste liquid during then modification of the flexible photoelectrode of *μ*PAD. In addition, to make for more friendly operation during this procedure, the reference lines are designed to accurately control the movement of detection tab of *μ*PAD, so as to easily accomplish the washing process by simply dragging the washing tab.

The assembly process of *μ*PAD from modification to the final PEC detection state is shown in Figure 1B. (a) The folding state of the paper-based device. In this condition, the detection tab is placed above the washing Table Since the hydrophobic zone can ensure that the liquid stays in the working zone for a longer time, it is conducive to obtaining better modification results. (b,c) After modification, the detection tab was moved to waste pool to realize cleaning process with the collection of waste liquid. In this case, the reference line should coincide with the lower edge of the detection tab to achieve the efficient waste liquid collection. When the cleaning is complete, the cleaning tab is dragged upwards, so that the detection tab can be tested for further modification. (d) When all modifications processes are completed, the *μ*PAD is in this state (d of Figure 1B) for the PEC detection. Electrolyte solution is added from working zone, and the hollow tab is used to realize the connectivity of three electrodes to realize PEC detection. The corresponding physical images mentioned above are shown in Figure S3.

## 3. Results and Discussion

### 3.1. Characterization of Obtained Samples

**Figure 2:**
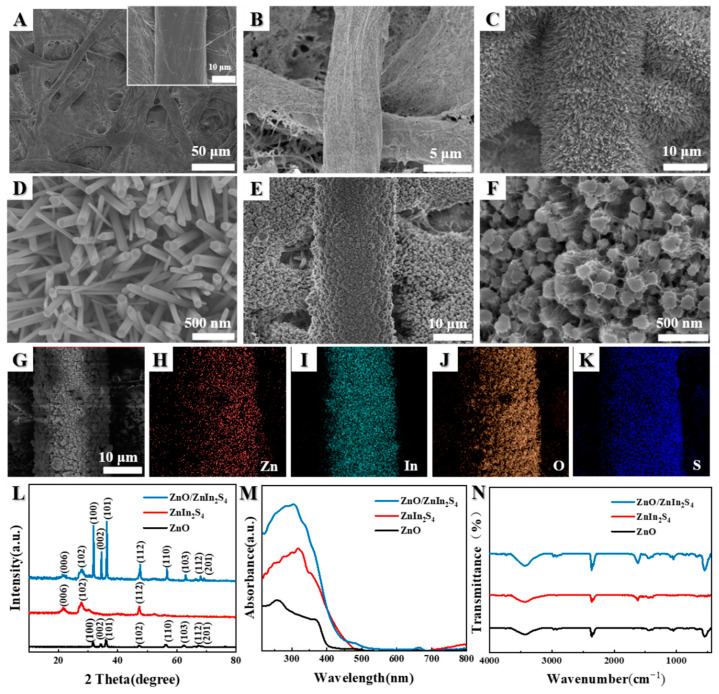
(**A**) SEM images of the bare paper fiber under different magnifications. (**B**) SEM image of Au/paper substrate. (**C**,**D**) SEM images of paper-based ZnO under different magnifications. (**E**,**F**) SEM images of paper-based ZnO/ZnIn_2_S_4_ under different magnifications. (**G**–**K**) The SEM-mode elemental mapping images for Zn, In, O, and S. (**L**) XRD of ZnO, ZnIn_2_S_4_ and ZnO/ZnIn_2_S_4_. (**M**) UV-vis data reaction system. (**N**) FT-IR spectra of ZnO, ZnIn_2_S_4_, and ZnO/ZnIn_2_S_4_.

In order to confirm the successfully synthesized, paper-based ZnO/ZnIn_2_S_4_ heterojunctions, the techniques include SEM, EDS, XRD, UV-vis, FT-IR, and XPS, which were applied. Firstly, the morphology of the prepared nanomaterials was characterized with the SEM technique. As indicated in Figure 2A, the large-scale, bare paper substrate showed a three-dimensional structure, with interconnecting cellulose fibers, offering a relatively high surface area for the modification of nanomaterials. The inset of Figure 2A depicted a single paper fiber of these thick and intricate cellulose fibers. To improve the conductivity of the paper fibers, a layer of Au NPs was modified to the surface of paper substrate. Figure 2B presented the gold nanoparticles modified on the cellulose fibers, with a relatively rough surface. After the electrodeposition, the orderly hexagonal NRs ZnO were uniformly growth onto the cellulose fibers with a high density of coverage, as shown in Figure 2C and D. The SEM images, with different scan ranges of ZnO/ZnIn_2_S_4_ heterojunction composites, are shown in Figure 2E and F, respectively. Observed with the magnified SEM image, the crystalline flake-like ZnIn_2_S_4_, composited with hexagonal NRs morphology of ZnO, is modified in situ onto the cellulose fibers surface of Au-PWE, in which crystalline flake-like ZnIn_2_S_4_ are almost fully covered on top of the hexagonal NRs ZnO, which makes it possible to provide more catalytical photoactive sites for PEC reaction and facilitate the access diffusion of reactants to biosensing interface, thus further promoting the PEC sensing performance. The successful synthesis of paper-based ZnO/ZnIn_2_S_4_ heterojunction, with the structure of a relatively large specific surface area, provides a guarantee for the construction of an immune biosensing platform. Whereafter, the elemental mapping under SEM mode was further employed for examining the distribution of Zn, O, In, and S in paper-based ZnO/ZnIn_2_S_4_ heterojunctions. As presented in Figure 2G–K, it is clearly shown that the ZnO/ZnIn_2_S_4_ composite was successfully synthesized, based on the presentation of the Zn, O, In, and S distributions. Furthermore, the results of EDS in Figure S4 demonstrated the presence of Zn, O, In, and S, which also validated the effective synthesis of ZnO/ZnIn_2_S_4_ heterojunctions. To further certify the crystalline texture and crystal phase composition of the obtained ZnO/ZnIn_2_S_4_ heterojunctions, XRD was carried out to characterize the pure ZnO, ZnIn_2_S_4_, and ZnO/ZnIn_2_S_4_ heterojunctions, as presented in Figure 2L. The characteristic peaks at 2θ = 31.8°, 34.8°, and 58.5° were orderly attributed to the (100), (101), and (110) planes diffractions of phase of ZnO. The characteristic peaks of pure ZnIn_2_S_4_ are at 2θ = 21.5°, 27.9°, and 47.3°, and they are attributed to the (006), (102), and (110) crystal planes of ZnIn_2_S_4_, which are consistent with the reported results in the literature [58]. Additionally, the XRD curve of ZnO/ZnIn_2_S_4_ heterojunctions is consistent with the XRD patterns of pure ZnO and ZnIn_2_S_4_, clearly indicating that the ZnO/ZnIn_2_S_4_ heterojunctions were successfully synthesized. To further study the synthesized ZnO/ZnIn_2_S_4_ heterostructures, UV-vis absorption spectra, and FT-IR spectra were also performed. As shown in Figure 2M, The UV-vis absorption spectrum of ZnO/ZnIn_2_S_4_ exhibits a broader range of absorption than that of ZnO, thus indicating that the ZnO/ZnIn_2_S_4_ heterojunctions achieved the enhanced light-harvesting ability. The FT-IR spectra obtained could be used to explore the structure vibrations and composition of the prepared composite, and the corresponding spectra of the synthesized composites are illustrated in Figure 2N in the scale of 440–4000 cm^−1^. The strong peaks at 3320, 1660, and 1388 cm^−1^, observed in all samples of ZnO, ZnIn_2_S_4_, and ZnIn_2_S_4_, can be attributed to the surface adsorbed water and hydroxyl group. In the spectra of pure ZnO, the peaks over the range of 600–400 cm^−1^ were attributed to the fundamental vibration of Zn–O [59]. In the FT-IR spectra of pure ZnIn_2_S_4_, the observed peaks were ascribed to the surface water molecules and hydroxyl groups [60]. Compared with the spectra curves in Figure 2N, the FT-IR peaks of ZnO/ZnIn_2_S_4_ heterojunctions were completely in accord with those of pure ZnO and ZnIn_2_S_4_, which further confirmed the successfully synthesized ZnO/ZnIn_2_S_4_ heterojunctions.

**Figure 3:**
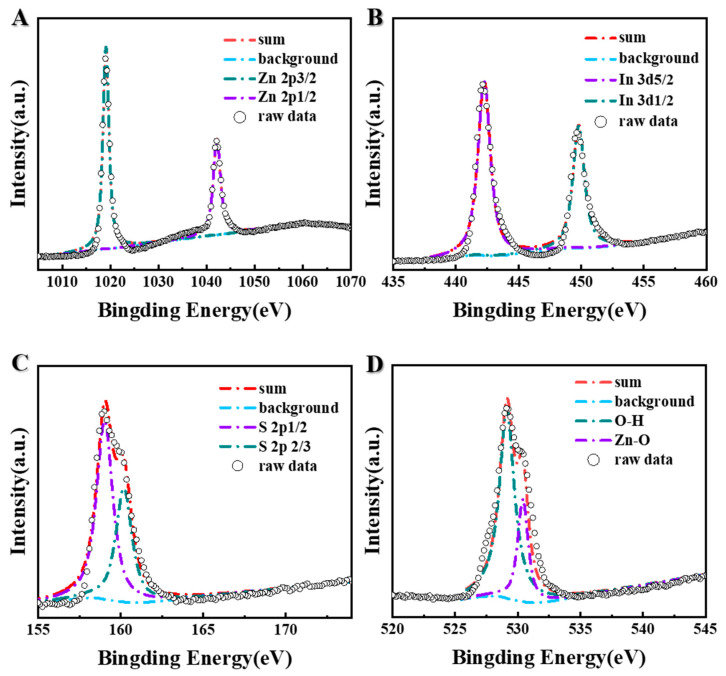
XPS spectrum of the ZnO/ZnIn_2_S_4_ heterojunctions: Zn 2p (**A**), In 3d (**B**), S 2p (**C**), and O 1s (**D**).

The XPS technique can be used for the identification of chemical states and the surface elemental composition of ZnO/ZnIn_2_S_4_ heterostructures. Figure S5 shows the primary binding energies of Zn_2p_, In_3d_, S_2p_, and O_1s_. As depicted in Figure 3, the diffraction peaks appeared at 1042.3 and 1019.1 eV are ascribed to the Zn_2p1/2_ and Zn_2p3/2_ orbitals of Zn^2+^ in ZnO/ZnIn_2_S_4_ heterostructure [61]. The In_3d_ orbitals are centered at 449.3 and 442.2 eV, ascribing to the In_3d3/2_ and In_3d5/2_ orbitals of In^3+^ in ZnIn_2_S_4_, as exhibited in Figure 3B [62]. The diffraction peaks positioned at 160.1 and 159.0 eV are ascribed, respectively, to the S_2p1/2_ and S_2p3/2_ orbitals of S^2^^−^ in ZnIn_2_S_4_, as presented at Figure 3C [63]. The O_1s_ peaks of the ZnO/ZnIn_2_S_4_ heterostructure are positioned at 530.2 and 529.2 eV, as shown in Figure 3D, which attributes to the lattice and adsorbed oxygen in ZnO, respectively [64].

### 3.2. Characterization of Photoelectric Properties of the Proposed μPAD

**Figure 4:**
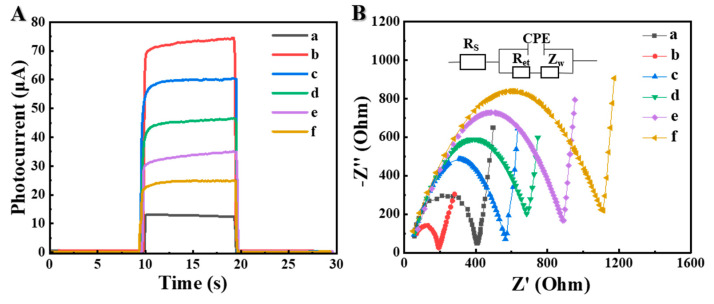
(**A**) PEC signals of the different modified photoelectrodes of *μ*PAD in PBS (0.1 M, pH 6.6) at 0.4 V: (a) paper-based ZnO, (b) ZnO/ZnIn_2_S_4_, (c) ZnO/ZnIn_2_S_4_/CS, (d) ZnO/ZnIn_2_S_4_/CS/Ab, (e) ZnO/ZnIn_2_S_4_/CS/Ab/BSA, and (f) ZnO/ZnIn_2_S_4_/CS/Ab/BSA/AFP (*c*_AFP_ = 0.1 ng mL^−1^). (**B**) EIS for different modification procedure in PBS (0.1 M, pH 6.6) including [Fe (CN)_6_] ^3-/4-^ (6.0 mM) and KCl (0.2 M): (a) paper-based ZnO, (b) ZnO/ZnIn_2_S_4_, (c) ZnO/ZnIn_2_S_4_/CS, (d) ZnO/ZnIn_2_S_4_/CS/Ab, (e) ZnO/ZnIn_2_S_4_/CS/Ab/BSA, and (f) paper-based ZnO/ZnIn_2_S_4_/CS/Ab/BSA/AFP (*c*_AFP_ = 0.1 ng mL^−1^).

To explore the PEC performance of the proposed *μ*PAD, the transient photocurrent was characterized by a chronoamperometry technique with a chopped light illumination. As depicted in Figure 4A, under the irradiation of a 500W xenon lamp, the PEC responses recorded from various modified photoelectrodes of the *μ*PAD, after completing several different modification steps, are investigated, with the on–off cycles at an adscititious potential of 0.4 V. For paper-based ZnO (a), 12.9 μA of photocurrent intensity is obtained. While forming ZnO/ZnIn_2_S_4_ heterojunctions with ZnIn_2_S_4_, the photocurrent is increased to 73.1 μA (b), which is 5.66 times stronger than that of paper-based ZnO, suggesting the integration of ZnIn_2_S_4_ into paper-based ZnO photoelectrode could notably improve the PEC performance, thus resulting from the built-in electric field of interface between ZnO and ZnIn_2_S_4_, which could further enhance the photoexcited electron/hole pairs conversion efficiency. After introducing biomolecules in each modification steps with CS, Ab, BSA, and AFP onto the paper-based ZnO/ZnIn_2_S_4_ photoelectrode, it is notable that the PEC response displays an obvious trend, with a gradual photocurrent signal response decline of paper-based ZnO /ZnIn_2_S_4_/CS (c), ZnO /ZnIn_2_S_4_/CS/Ab (d), ZnO /ZnIn_2_S_4_/CS/Ab/BSA (e), and ZnO /ZnIn_2_S_4_/CS/Ab/BSA/AFP (f), which is due to the relative insulative proteins and distinct steric hindrance from them in the PEC bioassay process. On the other hand, the gradual decline trend of photocurrent signals suggested that these different proteins/biomolecules in each constructing step were successfully modified onto the surface of paper-based ZnO /ZnIn_2_S_4_ photoelectrode. To further deliberate the construction procedure of the as-prepared *μ*PAD and properties of photogenerated charge carrier of relative paper-based photoelectrodes, the EIS method was performed in the phosphate buffer solution (PBS) (0.1 mol L^–1^, pH 6.6), including 6 mM [Fe (CN)_6_]^3–/4–^ as the redox probe, as shown in the Figure 4B. The electron transfers resistance (*Ret*) is expressed by observing the size of the semicircle diameter of the electrical impedance [65]. Interestingly, compared with the pure paper-based ZnO (b), the electron transfer rate of the sensing interface between ZnO/ZnIn_2_S_4_ is faster because of the formation of heterojunctions, based on the specific semicircle of paper-based ZnO/ZnIn_2_S_4_ (a) conjugate, which is smaller than paper-based ZnO. With the immobilization of CS (c), Ab (d), BSA (e), and AFP (f) in sequence onto the paper-based ZnO/ZnIn_2_S_4_ photoelectrode, the *Ret* increased gradually because each further modification of these biomolecules could impede the ferricyanide diffusion to the surface of the prepared photoelectrode. Hence, the displayed EIS curves of each modification process of the photoelectrode suggested the successful fabrication of the *μ*PAD for bioassays.

### 3.3. Optimization of Conditions for the μPAD

**Figure 5:**
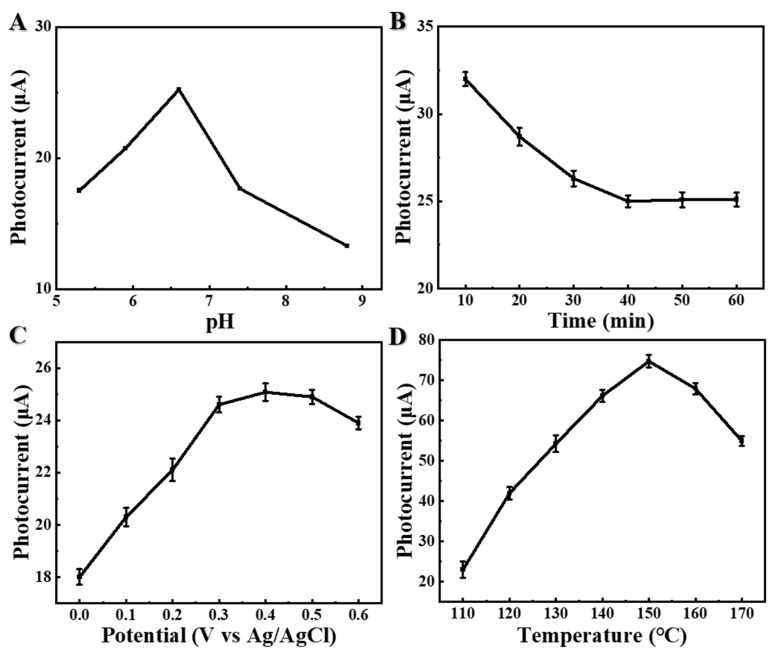
Effect of (**A**) the pH of PBS on photocurrent responses of the paper-based biosensor (*c*_AFP_ = 0.1 ng mL^−1^). (**B**) The incubation time of AFP (*c*_AFP_ = 0.1 ng mL^−1^). (**C**) The potential of PEC measurement (*c*_AFP_ = 0.1 ng mL^−1^). (**D**) The photocurrent of different synthesis temperature of ZnIn_2_S_4_ nanoflakes.

To achieve the optimum PEC performance for the proposed *μ*PAD, the experimental parameters, such as the PH value of prepared electrolyte, incubation time of AFP, potential of PEC performance, and synthesis temperature of ZnIn_2_S_4_ nanoflakes, have been fully measured. Firstly, Figure S6 exhibits the optimum PEC signals of paper-based ZnO, ZnIn_2_S_4_, and ZnO/ZnIn_2_S_4_. Obviously, the photocurrent responds of paper-based ZnO/ZnIn_2_S_4_ is strongly enhanced, compared with the paper-based ZnO and ZnIn_2_S_4_, thus indicating that the formation of this kind of type II heterostructures can facilitate the charge separation of interface, which is normally crucial for the improvement of the proposed PEC *μ*PAD performance. Then, the pH of electrolyte with different conditions were tested. As shown in Figure 5A, the intensity progressively increased with the increasing pH and arrived at an inflection point at pH 6.6. Thus, pH 6.6 is used for future experiment operation. As shown in Figure 5B, when the incubation time was within 40 min, the photocurrent response reduced gradually with incremental time. Afterward, as the incubation time exceeded 40 min, and the photocurrent intensity tended to be stabilized for the longer incubation time till 60 min. Hence, the 40 min was checked as the appropriate incubation time for AFP. From Figure 5C, the photocurrent intensity ascended from 0.1 to 0.4 V, decreased gradually with increasing potential, and reached 0.6 V. The maximum photocurrent is about 25.1 μA at 0.4 V, when the potential is within the range of 0–0.6 V. Thus, the optimal potential of 0.4 V was determined to achieve the detection of AFP. Finally, the different synthesis temperatures of crystalline flake-like ZnIn_2_S_4_ could influence the photocurrent intensity of the photoelectrode. As shown in Figure 5D, when the synthesis temperature of crystalline flake-like ZnIn_2_S_4_ was within 150 °C, the current response enhanced gradually with the rising synthesis temperature of ZnIn_2_S_4_ nanoflakes. However, when the synthesis temperature of ZnIn_2_S_4_ was further elevated to 170 °C, the photocurrent would decrease with the increasing synthesis temperature of ZnIn_2_S_4_ nanoflakes. Therefore, the 150 °C was selected as the optimal synthesis temperature of crystalline flake-like ZnIn_2_S_4_ and used in the further investigations.

### 3.4. Specific Performance of the μPAD of PEC for AFP Immunosensing

**Figure 6:**
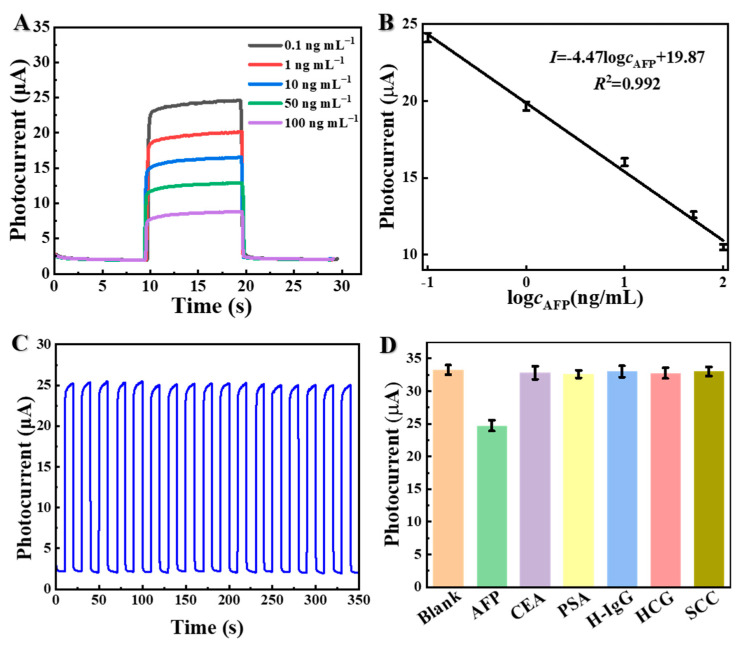
(**A**) PEC signals of different concentrations of AFP. (**B**) Calibration curve of photocurrent vs logarithm of AFP concentration. (**C**) Stability of the PEC signals for 350 s (*c*_AFP_ = 0.1 ng mL^−1^). (**D**) Selectivity of the proposed PEC assay of AFP (*c*_AFP_ = 0.1 ng mL^−1^). Error bars reflect the standard deviation of the measured data of three parallel experiments. All PEC signals were performed in 0.1 M PBS (pH 6.6) containing 0.1 M AA, and the applied potential was set at 0.4 V.

To demonstrate the feasibility of the fabricated PEC *μ*PAD for AFP detection, the photocurrent response of it was measured for different AFP incubation concentrations. Figure 6A presents the PEC signals under the optimum condition, after incubation using a series of concentrations of AFP. With the addition of the concentration of AFP from 0.1 to 100 ng mL^−1^, more AFP targets would be progressively acquired onto the biosensing interface of the detecting zone of the *μ*PAD assistant, while the specifically recognizing the interaction of Ab and accordingly introducing a weakened photocurrent tendency of the PEC signals output, due to the apparent steric effect and inhibition of AA to contact with paper-based photoelectrode surface, further realizing the quantitative sensing of AFP targets. As described in Figure 6B, the matching calibration plot displayed that a progressive decline of the PEC photocurrent intensity was explicitly proportional to the concentration logarithmically of AFP within the range of 0.1–100 ng mL^−1^. The resulting linear correlation equation is *I* = −4.47 log *c*_AFP_ + 19.87 (R^2^ = 0.992), with the corresponding limit of detection (LOD) of 0.03 ng mL^−^^1^ and a signal-to-noise ratio of 3. Compared with the previously reported works (Table S1: comparison with other PEC-based AFP biosensors), the proposed PEC immunosensor presented a wide detection and low LOD, which attributed to the in-situ grown ZnO/ZnIn_2_S_4_ heterojunctions onto cellulose fibers possessing good PEC activity, thus indicating the proposed biosensor has further application potential in clinical analysis. Furthermore, a comparison of the analytical performances for the detection of various AFP different immunological methods is summarized in Table S2. Obviously, it can be seen that the proposed immunosensor we prepared has a superior sensibility and acceptable detection range. Figure 6C shows the stability of the *μ*PAD on the premise of optimization conditions. For the measurement, electrolyte solution is added from working zone of the paper-based biosensor after incubation of AFP for 40 min. Then, the working voltage was set to 0.4 V, and the hollow tab is used to realize the connectivity of three electrodes to realize PEC detection. After the current was stable, the working electrode was irradiated, and the light was switched on and off manually at a period of 10 s. The PEC signals were recorded as the photo illumination was turned on and off for 10 s, about 17 cycles. Record 350 s later, the change of PEC signal was inconspicuous, compared with its initial photocurrent value, suggesting the good photostability of the proposed PEC *μ*PAD. As for the ones depicted in Figure 6D, the photocurrent of AFP, carcinoembryonic antigen (CEA), prostate-specific antigen (PSA), human immunoglobulin G (H-IgG), human chorionic gonadotropin (HCG), and squamous cell carcinoma antigen (SCC) were tested to assess the specificity of the prepared biosensor. AFP, CEA, PSA, H-IgG, HCG, and SCC are common serum tumor markers [66,67]. Serum tumor markers refer to proteins in the form of carbohydrate antigens, hormones, receptors, enzymes or metabolites, oncogenes, tumor suppressor genes, and their related products; those increase with the appearance of tumors. These biomolecules are produced by tumor cells and secreted into the serum and detected, which can reflect the presence of tumors in the body, to a certain extent [68]. Only the presence of AFP target causes the intuitive attenuation of PEC signals, and all those interferential reagents are alike to the results of the blank group, with a marked increase in the results of the AFP group, thus implying satisfactory and specific selectivity of the as-prepared *μ*PAD to detect AFP via the signal-off PEC readout strategy. Specifically, the relative standard deviation (RSD) was calculated to be less than 2%, indicating the credible of the constructed biosensor. In addition, to study the influence of longer storage time for the *μ*PAD, the PEC signals of the as-prepared sensor stored at 4 ℃ were performed every 4 days after incubation with 0.1 ng mL^−1^ AFP (Figure S7). After 24 days, the optimum PEC intensity generated by the PEC *μ*PAD could also reach 91.85% of the initial intensity, which demonstrated that the as-prepared PEC *μ*PAD has a high reliability.

### 3.5. Actual Samples Analysis

To investigate applicability of the proposed PEC *μ*PAD toward real sample, the clinical serum samples were detected. More specifically, real serum samples from the whole blood of healthy people were collected by the operation of centrifugation (11000 rpm, 12 min), so that the practicality and dependability of the proposed *μ*PAD to detect AFP could be verified. The samples were diluted, and the different AFP concentrations of the samples were added by standard method. The experimental results (the average of five parallel experimental findings) are summarized clearly in Table 1. The RSD of AFP detection varied from 1.21% to 5.37%, and the AFP recovery rate ranged between 95.2% and 100.8%, demonstrating that the proposed PEC could be further applied for the clinical diagnosis of detecting AFP.

## 4. Conclusions

In summary, this work was the first to demonstrate cellulose paper fibers mediated in situ grown ZnO/ZnIn_2_S_4_ heterojunctions for PEC immunoassay of AFP targets. The synthesized ZnO/ZnIn_2_S_4_ heterojunctions with crystalline flake-like ZnIn_2_S_4_ modified with hexagonal NRs ZnO onto paper fibers was characterized and confirmed via different techniques, including SEM, EDS, XRD, UV-vis, FT-IR spectroscopy, XPS, and PEC measurements. Combined with a delicate design of *μ*PAD, the evenly in situ generated ZnO/ZnIn_2_S_4_ heterojunctions onto Au-PWE as a transducer material that remarkably reduced the background of signal response and extended the absorption of light resource. Consequently, a novel PEC immunoassay for AFP targets sensing platform was successfully achieved, with the advantage of high sensitivity, good selectivity, and reproducibility, due to the effective photoexcited electron-hole separation transfer path from the proposed composites onto paper fibers. The presented strategy confirmed the successful feasibility of the PEC sensing system for the possibility of the rapid and efficient prediction of diseases, based on the sensitive detection of AFP targets, and could pave a promising way for the construction of paper-based biosensing platform in next-generation clinical diagnosis applications.

## Figures and Tables

**Table 1:** Assay results of real human serum by proposed *μ*PAD.

Serum Sample	Added, ^a^ (ng mL^−1^)	Found, (ng mL^−1^)	Recovery, ^b^ %	RSD, %
1	0.1	10.08 × 10^−2^	100.8	5.02
2	0.5	4.90 × 10^−1^	98.0	5.37
3	1	9.77 × 10^−1^	97.7	3.29
4	5	4.76	95.2	2.80
5	10	9.59	95.9	1.21

^a^ AFP standards with different concentrations were added into the corresponding initial human serum samples. ^b^ The recovery was estimated by the value (the found concentration subtracted that of PEC immunoassay), relative to the addition of AFP amount.

## Supplementary Materials

The following supporting information can be downloaded at: https://www.mdpi.com/article/10.3390/bios12100818/s1. The details of preparation of Au/paper substrate; modification process of the working electrode. Table S1: Comparison of other PEC-based AFP biosensors; Table S2: Comparison of methods for the detection of AFP; Figure S1: Wax-patterns of the *μ*PAD on a paper sheet (A4); A: before baking; B: after baking. Figure S2: Printing of electrode of the *μ*PAD on a paper sheet (A4). Figure S3: The physical picture of modification and detection of *μ*PAD. Figure S4: EDS spectrum of ZnO/ZnIn_2_S_4_. Figure S5: XPS spectra of ZnO/ZnIn_2_S_4_ wide scan. Figure S6: Photocurrent of ZnO, ZnIn_2_S_4_, and ZnO/ZnIn_2_S_4_. Figure S7: Stability of the proposed biosensor for different periods of storage. References [69,70,71,72,73,74,75] are cited in the supplementary materials.
