# Supplementary material for: A Paper-Based Photoelectrochemical Sensing Platform Based on In Situ Grown ZnO/ZnIn2S4 Heterojunctions onto Paper Fibers for Sensitively Detecting AFP
Source: Biosensors (Basel). 2022 Oct 2;12(10):818. doi: 10.3390/bios12100818 (PMC9599276; doi:10.3390/bios12100818)
Supplement: Supplementary file 1 [file biosensors-12-00818-s001.zip › biosensors-1902461-supplementary.pdf]

Supporting Information

A paper-based photoelectrochemical sensing platform based on in situ grown $ZnO/ZnIn_2S_4$ heterojunctions onto paper fibers for sensitively detecting AFP

Jiali Huang [1,†], Xu Li [1,†], Mingzhen Xiu [2], Kang Huang [2], Kang Cui [1,2,*], Jing Zhang [1], Shiji Hao [4], Shenguang Ge [1,3,*], Jinghua Yu [1], Yizhong Huang[2,*]

[1] School of Chemistry and Chemical Engineering, University of Jinan, Jinan 250022

[2] School of Materials Science and Engineering, Nanyang Technological University, Singapore 639798, Singapore

[3] Institute for Advanced Interdisciplinary Research, University of Jinan, Jinan 250022, P. R. China

[4] School of Materials Science & Engineering, Dongguan University of Technology, Guangdong 523808, P. R. China

* Correspondence: chm_cuik@ujn.edu.cn (K. C.); chm_gesg@163.com (S. G.); yzhuang@ntu.edu.sg (Y. H.);

† These authors contributed equally to this work.

Content

Preparation of Au nanoparticle modified paper working electrode (Au-PWE)....1

Modification process of the working electrode 1

Table S1 Comparison of other PEC-based AFP biosensors 2

Table S2 Comparison of methods for the detection of AFP 2

Figure S1. Wax-patterns of the μPAD on a paper sheet (A4). (A): before baking; (B): after baking. 3

Figure S2. Printing of electrode of the μPAD on a paper sheet (A4). (A): The side of the A4 paper that is printed with carbon electrode; (B): The reverse side of Figure S2A. 3

Figure S3. The physical picture of modification and detection of μPAD....................4

Figure S4. Energy-dispersive spectrum (EDS) spectrum of $ZnO/ZnIn_2S_4$..................4

Figure S5. XPS spectra of $ZnO/ZnIn_2S_4$ wide scan...5

Figure S6. Photocurrent responds of ZnO, $ZnIn_2S_4$ and $ZnO/ZnIn_2S_4$........................5

Figure S7. Stability of the proposed μPAD for different periods of storage: 0 days, 4 days, 8 days, 12 days, 16 days, and 24 days (c_{AFP} = 0.1 ng mL^{-1})................................5

References..6

Preparation of Au nanoparticle modified paper working electrode (Au-PWE)

The Au/paper substrate of μPAD was obtained by growing Au NPs layer on the cellulose fibers. Herein, 80 mL of ultrapure water was put into a round-bottom flask and heated it to 90 °C, and 0.8 ml of 1% $HAuCl_4$ solution was dripped to the prepared solution and then heated to 96 °C for 1 min. Next, 2.8 ml of 1% trisodium citrate was added and stirred for 20 min. Finally, the above solution was stirred and cooled to receive a gold seed solution. After that, 80 μL of hydroxylamine hydrochloride and gold seed solution (volume ratio 1:1) was dropped on the surface of Au/paper substrate each time and dried at air. After repeated dripping five times, it was thoroughly washed with ultrapure water and dried at 60 °C. The Au-PWE was prepared successfully.

Modification process of the working electrode

Firstly, chitosan solution (0.1 wt.%, 20 μL) was dropped on the surface of photoelectrode and left to dry at 4 °C for 24 h, followed by washing with 0.01 M NaOH and ultrapure water. After that, glutaraldehyde solution (5 wt.%, 25 μL) was spread onto the photoelectrode, keeping for 35 min and washed thoroughly with ultrapure water. The antibody solution (100 μg/mL, 30 μL) was dropped onto the activated photoelectrode. After being incubated at room temperature for 24 h, the activated photoelectrode was washed with PBS. After that, 25 μL of 0.2% bovine serum albumin (BSA) solution dissolved by PBS (0.1 mol L^{-1}, pH 6.6) was added on the antibody modified photoelectrode for 1 h at 4 °C to block non-specific binding sites. Finally, 30 μL antigen of AFP with different concentration were added onto the photoelectrode and incubated at 4 °C for 40 min, followed by washing thoroughly with PBS. Then, the signals of photocurrent were measured on the CHI 660C electrochemical working station in PBS solution (0.1 mol L^{-1}, pH 6.6) dissolved 0.1 M ascorbic acid. During the PEC signal measurement procedure, a PLS-SXE 300 xenon lamp (500 W) was utilized as the irradiation source, which was switched on and off every 10 s and the applied potential was 0.4 V.

Table S1 Comparison of other PEC-based AFP biosensors

Materials	Detection range (ng mL^{-1})	LOD (ng mL^{-1})	Ref.
β-In_2S_3@carbon dots	0.5-50	0.0379	[1]
graded oxygen-doped CdS	0.2-100	0.0784	[2]
MoS_2/Au/GaN	1.0-150	0.3	[3]
RGO/dimethyldioctadecylammonium/ZnO	0.05-100	0.05	[4]
ZnO/$ZnIn_2S_4$	0.1-100	0.03	This work

Table S2 Comparison of methods for the detection of AFP

Analytical method	Detection range (ng mL^{-1})	LOD (ng mL^{-1})	Ref.
Fluorescence immunoassay	1-80	0.45	[5]
Electrochemical immunoassay	0.5-50	0.1	[6]
Chemiluminescent immunosensor	5-70	2.5	[7]
PEC immunosensor	0.1-150	0.1	[8]
PEC immunosensor	0.1-100	0.03	This work

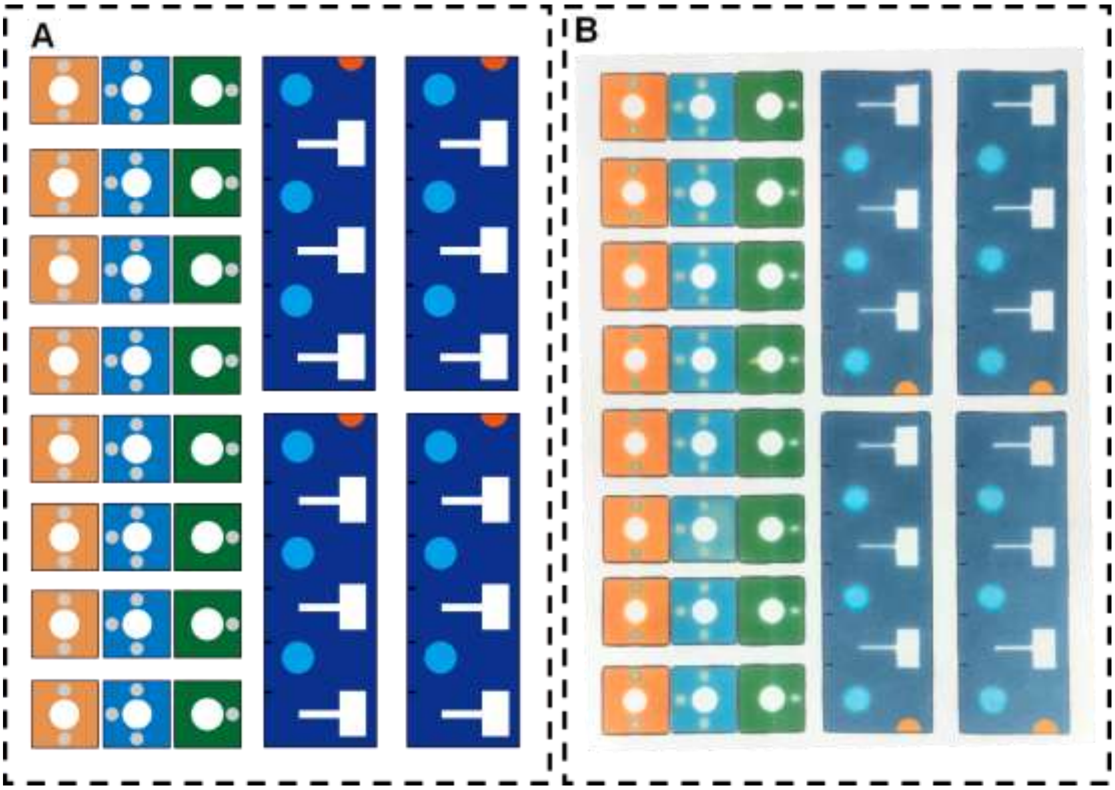

Figure S1. Wax-patterns of the μPAD on a paper sheet (A4). (A): before baking; (B): after baking.

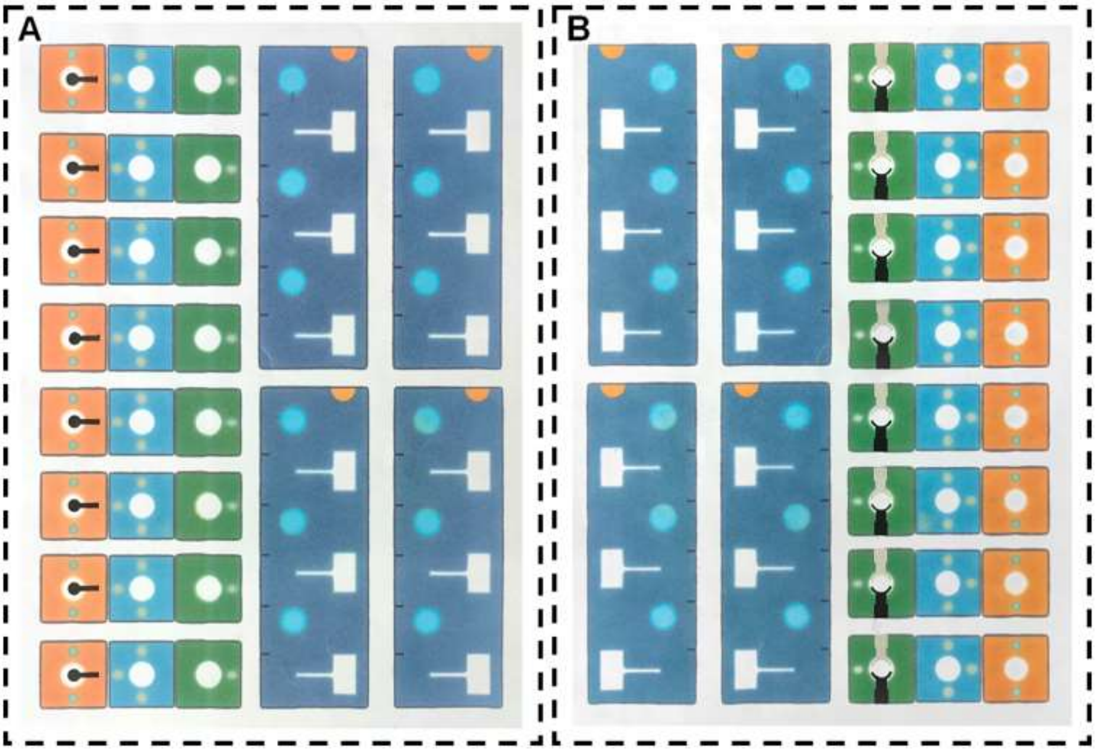

Figure S2. Printing of electrode of the μPAD on a paper sheet (A4). (A): The side of the A4 paper that is printed with carbon electrode; (B): The reverse side of Figure S2A.

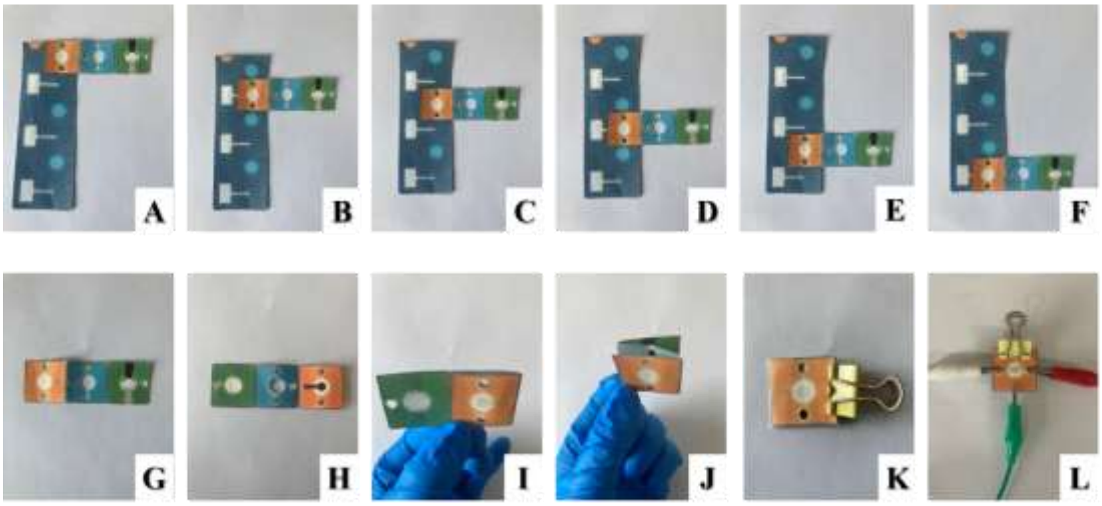

Figure S3. The physical picture of modification and detection of μPAD. (A), (C), and (E): The μPAD during the process of electrode modification; (B), (D), and (F): The washing process of the detection tab; (G) and (H): Unfolded μPAD; (I) and (J): The folding process of the μPAD; (K) and (L): The μPAD fold state during the process of PEC.

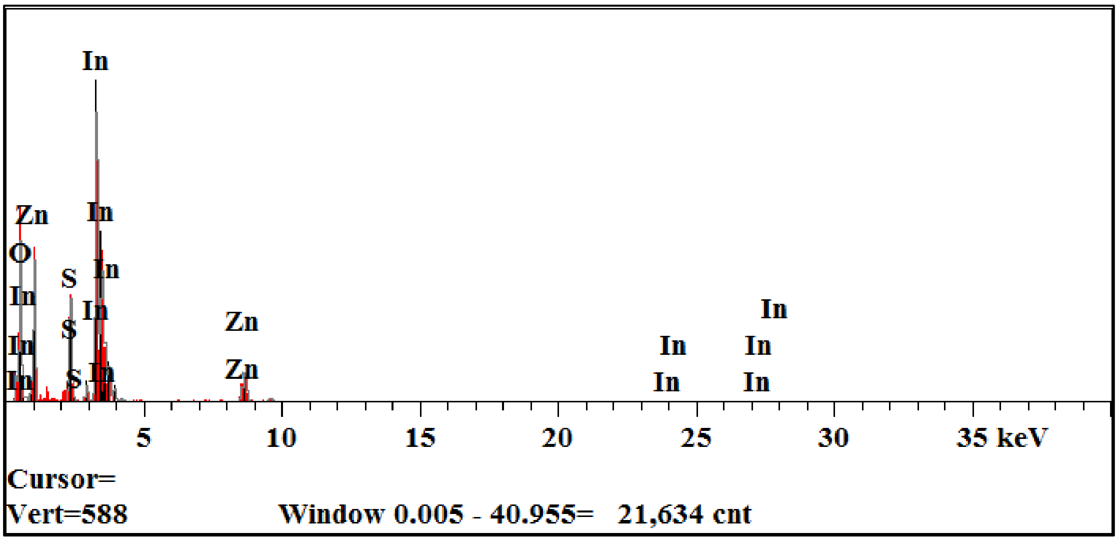

Figure S4. Energy-dispersive spectrum (EDS) spectrum of $ZnO/ZnIn_2S_4$.

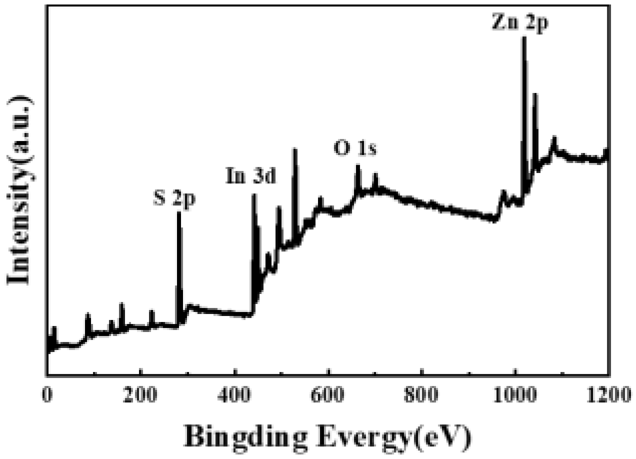

Figure S5. XPS spectra of $ZnO/ZnIn_2S_4$ wide scan.

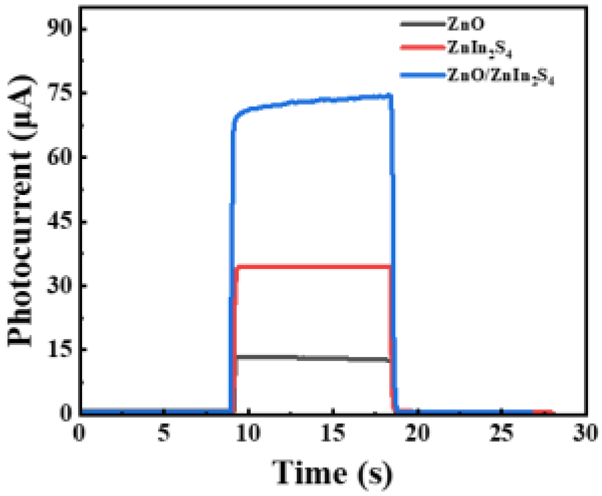

Figure S6. Photocurrent responds of ZnO, $ZnIn_2S_4$ and $ZnO/ZnIn_2S_4$. The PEC measurement was performed in the PBS (0.1 M, pH 6.6) containing 0.1 M AA, and the applied potential was 0.4 V.

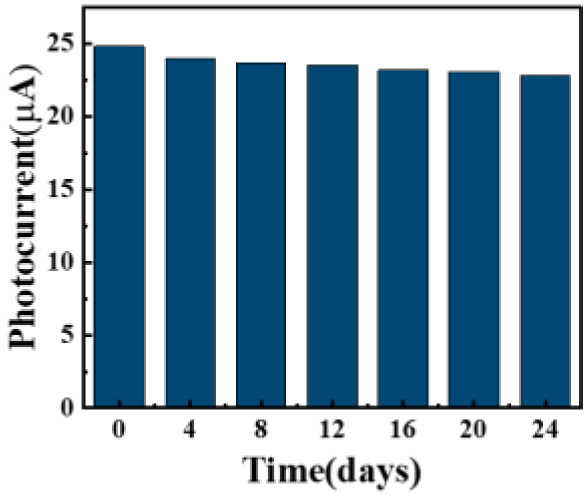

Figure S7. Stability of the proposed μPAD for different periods of storage: 0 days, 4 days, 8 days, 12 days, 16 days, and 24 days (c_{AFP} = 0.1 ng mL^{-1}). The PEC measurement was performed in the PBS (0.1 M, pH 6.6) containing 0.1 M AA, and the applied potential was 0.4 V.

References

1. Yu, Z., Huang, L., Chen, J., Tang, Y., Xia, B. and Tang, D., Full-Spectrum Responsive Photoelectrochemical Immunoassay Based on B-In_2S_3@Carbon Dot Nanoflowers. *Electrochim. Acta* **2020,** *332*, 135473.
2. Yu, Z., Huang, L., Chen, J., Li, M. and Tang, D., Graded Oxygen-Doped CdS Electrode for Portable Photoelectrochemical Immunoassay of Alpha-Fetoprotein Coupling with a Digital Multimeter Readout. *Sens. Actuators B Chem.* **2021,** *343,*130136.
3. Hu, D., Cui, H., Wang, X., Luo, F., Qiu, B., Cai, W., Huang, H., Wang, J. and Lin, Z., Highly Sensitive and Selective Photoelectrochemical Aptasensors for Cancer Biomarkers Based on MoS_2/Au/GaN Photoelectrodes. *Anal. Chem.* **2021,** *93*, 7341-7347.
4. Wu, Y., Su, H., Yang, J., Wang, Z., Li, D., Sun, H., Guo, X. and Yin, S., Photoelectrochemical Immunosensor for Sensitive Detection of Alpha-Fetoprotein Based on a Graphene Honeycomb Film. *J. Colloid Interface Sci.* **2020,** *580*, 583-591.
5. Wang, Y., Chen, L., Liang, M., Xu, H., Tang, S., Yang, H. and Song, H., Sensitive fluorescence immunoassay of alpha-fetoprotein through copper ions modulated growth of quantum dots in-situ. *Sens. Actuators B Chem.* **2017,** *247*, 408-413.
6. Kong, F., Xu, B., Du, Y., Xu, J. and Chen, H., A branched electrode based electrochemical platform: towards new label-free and reagentless simultaneous detection of two biomarkers. *Chem. Commun.* **2013,** *49*, 1052-1054.
7. Huang, X. and Ren, J. Gold nanoparticles based chemiluminescent resonance energy transfer for immunoassay of alpha fetoprotein cancer marker. *Anal. Chim. Acta.* **2011,** *686*, 115-120.
8. Liu, X., Song, X., Dong, Z., Meng, X., Chen, Y., Yang, L., Photonic crystal fiber-based immunosensor for high-performance detection of alpha fetoprotein. Biosens. Bioelectron. **2017,** *91*, 431-435.
